# Regulator of G-Protein Signalling-14 (RGS14) Regulates the Activation of αMβ2 Integrin during Phagocytosis

**DOI:** 10.1371/journal.pone.0069163

**Published:** 2013-06-21

**Authors:** Jenson Lim, Jo Thompson, Robin C. May, Neil A. Hotchin, Emmanuelle Caron

**Affiliations:** 1 School of Biosciences, University of Birmingham, Edgbaston, Birmingham, United Kingdom; 2 nanoTherics Ltd., Newcastle under Lyme, Staffordshire, United Kingdom; 3 Royal Devon and Exeter Hospital, Exeter, Devon, United Kingdom; 4 Centre for Molecular Microbiology and Infection and Division of Cell and Molecular Biology, Imperial College London, London, United Kingdom; University of Iowa, United States of America

## Abstract

Integrin-mediated phagocytosis, an important physiological activity undertaken by professional phagocytes, requires bidirectional signalling to/from αMβ2 integrin and involves Rap1 and Rho GTPases. The action of Rap1 and the cytoskeletal protein talin in activating αMβ2 integrins, in a RIAM-independent manner, has been previously shown to be critical during phagocytosis in mammalian phagocytes. However, the events downstream of Rap1 are not clearly understood. Our data demonstrate that one potential Rap1 effector, Regulator of G-Protein Signalling-14 (RGS14), is involved in activating αMβ2. Exogenous expression of RGS14 in COS-7 cells expressing αMβ2 results in increased binding of C3bi-opsonised sheep red blood cells. Consistent with this, knock-down of RGS14 in J774.A1 macrophages results in decreased association with C3bi-opsonised sheep red blood cells. Regulation of αMβ2 function occurs through the R333 residue of the RGS14 Ras/Rap binding domain (RBD) and the F754 residue of β2, residues previously shown to be involved in binding of H-Ras and talin1 head binding prior to αMβ2 activation, respectively. Surprisingly, overexpression of talin2 or RAPL had no effect on αMβ2 regulation. Our results establish for the first time a role for RGS14 in the mechanism of Rap1/talin1 activation of αMβ2 during phagocytosis.

## Introduction

Phagocytosis has a critical physiological function as part of the feeding process in amoebae, or as part of the innate immune system which functions to remove microorganisms and apoptotic cells in mammals [1]. In humans the process of phagocytosis is undertaken by professional phagocytes such as neutrophils and macrophages. Phagocytosis involves receptor-mediated particle recognition, actin-driven uptake, phagosome maturation and particle clearance. Surface-expressed phagocytic receptors exist that can bind their target directly or indirectly through opsonins, depending on cell type and the nature of its targets [2]. Two well characterized phagocytic receptors are the Fcγ receptor (FcγR) and complement receptor 3 (aka CR3, Mac-1, αMβ2, CD11b/ CD18), that bind IgG- or C3bi-opsonised particles, respectively [1,2].

CR3 is an integrin, comprised of a single αM and a single β2 subunit. Integrin subunits are made up of a large extracellular ligand-binding domain, a single pass transmembrane domain and a short cytoplasmic tail and they are bi-directionally regulated. “Inside-out” activation of αMβ2 involves the cytoskeletal protein, talin, the small GTP-binding protein, Rap1, and calcium/calmodulin kinase II and leads to particle binding [3–6]. Association with a complement-opsonised particle leads to “outside-in” signalling which ultimately leads to the RhoA-dependent uptake, and subsequent destruction, of the particle [7,8].

Previously, we established both that Rap1 action was upstream of talin and that exposure of talin1 head domain was crucial for “inside-out” activation of αMβ2 during phagocytosis in macrophages [4,5]. This signalling pathway occurs independent of **RIAM**, a well-documented Rap1 effector crucial for αIIbβ3 integrin function [9]. Several other potential Rap1 effectors have been identified, although most of these also bind to other Ras GTPases in vitro – e.g. **Vav2**, Regulator for cell Adhesion and Polarization enriched in Lymphoid tissues (RAPL), Afadin/MLLT4 (**AF6**) and Regulator of G-Protein Signalling-14 (**RGS14**) [10–13]. Others, like Phosphatidylinositol 3-kinases, RalA and Raf have been dismissed (Caron, unpublished data) [14–16]. Recent work has also identified other FERM-containing proteins, such as talin2 and kindlin3, the latter of which has a positive role in integrin activation [17,18]. The aim of this study was to identify the Rap1 effector involved in activation of αMβ2 and to establish the role, if any, of talin2 in this process. We show that the Rap1 effector, RGS14 regulates αMβ2, and that this regulation is dependent on R333 of RGS14 and F754 of β2. However, we find no evidence that Talin2 is involved in this process.

## Methods

### Materials

Sheep red blood cells (RBC) were purchased from TCS Biosciences, Ltd., Gelatin Veronal Buffer, and C5-deficient serum were from Sigma. The antibodies used in this study were mouse anti-RGS14 (clone H-70, Santa Cruz), mouse anti-human β2 (clone 6.7; BD-Pharmingen), mouse anti-Flag (M2, Sigma), mouse anti-myc (9E11, Cell Signalling) rabbit anti-FAK (clone Ab-397, Sigma) and rabbit anti-sheep erythrocyte IgM antibodies (Cedarlane Laboratories). Conjugated secondary antibodies were from Molecular Probes/Invitrogen (immunofluorescence) or IRDye (western blots).

### DNA constructs

Eukaryotic expression vectors (pRK5) encoding human wild type (wt) and mutant αM and β2 were previously described [4,6–8]. Plasmids used in this study are as follows: pRKGFP-Talin1 (Kazue Matsumoto), pEGFP-Talin2 [17], pCDNA3.1myc-AF6 (Linda Van Aelst), pCMV3myc-Vav2 [10], pCDNA3.1Flag -RGS14, -RGS14(H406A) and -RGS14(R333L) [19], pCDNA4myc-RAPL [20]. All plasmids were transformed into One Shot TOP10 chemically competent *Escherichia coli* (Invitrogen) and DNA was prepared using the QIAGEN maxi- or mini-prep kits.

### Cell culture and transfection

Cells from the murine macrophages J774.A1 and simian kidney fibroblast COS-7 (American Type Culture Collection numbers TIB-67 and CRL-1651, respectively) were maintained and seeded as previously described [7]. Transfection of COS-7 and J774.A1 cells with plasmid DNA or siRNA were performed using Genejuice (Merck Millipore) or RNAiMAX (Invitrogen), respectively, according to manufacturers’ instructions. For gene knockdown of RGS14, J774.A1 cells were transfected with 60pmol ON-TARGET *plus* siRNA (individual or a pool of four, Dharmacon/Thermo Scientific) and allowed to undergo gene silencing for 5 days. For transfection of J774.A1 cells with plasmid DNA, magnetofection using the magnefect-nano II system (nanoTherics Ltd, Stoke-on-Trent, UK) was employed. Briefly, 1.5μg of plasmid DNA was complexed with 1μl of Neuromag (nanoTherics Ltd) for 15min in serum-free DMEM before adding drop-wise to 10 000 non-activated J774.A1 cells per well in 24-well plate. Plates were incubated for 30min over a magnet array moving laterally at 2Hz with 0.2mm amplitude of displacement (oscillating). After transfection, plates were removed from the magnetic arrays and placed back in the incubator. 24hr later, the same procedure was repeated (retransfection) and cells were incubated for a further 24hr before phagocytic challenge or lysed with sample buffer for western blotting. Intensities of bands were determined by densitometric analysis by using the ImageJ software (National Institutes of Health) and related to the levels of Scrambled siRNA/FAK loading control.

### Phagocytic challenge

C3bi-opsonised RBC (C3bi-RBC) were prepared and used as previously described [7,8], using 0.1μl (0.5μl for macrophages) of fresh RBC per 13mm coverslip. Where needed for efficient binding and phagocytosis of C3bi-opsonised RBC, macrophages were preactivated using 150ng/ml phorbol-12-myristate-13-acetate (PMA, Sigma) in HEPES-buffered, serum-free DMEM for 15min at 37°C [21]. PMA-activated macrophages were challenged with C3bi-RBC for 30min at 37°C, washed with PBS to remove unbound RBC and fixed in cold 4% paraformaldehyde for 10min at 4°C.

### Immunofluorescence and scoring

Since only associated RBC were counted, J774.A1 or COS-7 cells were permeabilised with 0.1% Triton X-100 and stained for either β2 (1:100 dilution), myc- or Flag- (both at 1:1000 dilution) tagged proteins before counterstained using Alexa-Fluor-488-conjugated goat anti-mouse (GFP-tagged proteins remained unstained). To highlight the C3bi-SRBC, the Alexa-Fluor-594-conjugated goat anti-rabbit antibodies were applied, which would be against the opsonising rabbit antibodies. Coverslips were finally mounted in Mowiol (Calbiochem) containing *p*-phenylenediamine (Sigma) as antifading reagent and analyzed by confocal or epifluorescence microscopy (LSM710, Zeiss or DM-IL, Leica). Association index is defined as the number of RBC bound to 100 macrophages or COS-7 cells expressing appropriate plasmids. Data shown are the mean ± SEM of at least three independent experiments.

### Statistical analysis

One-way analysis of variance (ANOVA) was used to compare and determine statistical significance (GraphPad InStat software). *P* values of less than 0.05 were considered significant.

## Results and Discussion

**Figure 1:**
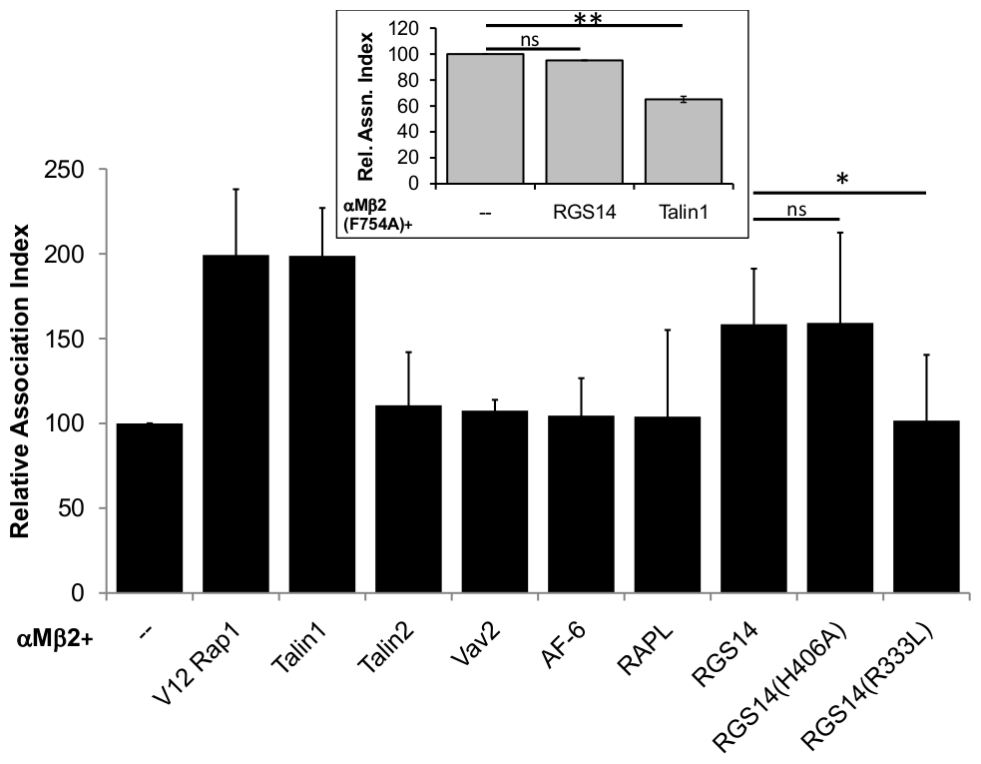
Identification of the Rap1 effector necessary for αMβ2 activation. COS-7 cells were co-transfected with wild type αM and β2 (main) or αM and β2(F754A) (inset), together with the Rap1 effectors, constitutively active Rap1 (V12), talins 1 and 2, as indicated. Transfected cells were challenged with C3bi-RBC, processed for immunofluorescence using the appropriate antibodies against either β2, myc- and Flag-tagged proteins or unstained for GFP-tagged proteins and scored for C3bi-RBC association, as described in the Methods section. Results were expressed relative to the values obtained for αMβ2 only (arbitrarily set to 100). Not significant (ns), *p*>0.05; *, *p*<0.05; **, *p*<0.001.

To identify the Rap1 effector required for activation of αMβ2, plasmids expressing known Rap1 effectors were transiently transfected into COS-7 cells together with wild type (wt) αM and β2 chains. After 48hr, the transfected cells were challenged with C3bi-opsonised sheep red blood cells (C3bi-RBC), immunostained and scored for associated C3bi-RBCs relative to COS-7 expressing only wt αMβ2 (Figure 1). Consistent with our previous findings, binding of C3bi-RBC increased by twofold to cells expressing αMβ2 together with either constitutively active Rap1 (V12) or full length talin1 [5]. Co-expression with Vav2, AF-6, talin2 or RAPL resulted in no increase in C3bi-RBC binding. However, co-expression of RGS14 with αMβ2 resulted in an increase in binding (158.5±32.7, *p*<0.05), though not to levels seen with either V12Rap1 (199.2±38.7, *p*<0.05) or talin1 (198.8±28.2, *p*<0.05). RGS14 was first identified as a Rap1 effector in a yeast two hybrid screen, using a mouse brain cDNA library, together with wt and point mutants of Rap1 and Rap2. This was confirmed using His-tagged RGS14 to pull down endogenous Rap1 from mouse brain membranes [13]. Therefore it is likely that RGS14 is the primary Rap1 effector responsible for activating αMβ2.

Next we mapped the regions of RGS14 and αMβ2, required for αMβ2 activation. RGS14 contains two Ras/Rap-binding domains (RBD), R1 and R2 which contain key residues critical for interacting with Ras and Rap isoforms – R333 (in R1) and H406 (in R2) [19,22]. Using point mutants of RGS14 (R333L and H406A), we found that the H406A mutant of RGS14 (H406A = 157.9±47.6 c.f. wt RGS14 = 160.3±31.4; *p*>0.05), but not the R333L mutant (R333L = 98.6±35.4 c.f. wt RGS14 = 160.3±31.4; *p*<0.05), was able to activate αMβ2 comparable with wt RGS14 (Figure 1). This finding indicates that it is the R1 RBD that is crucial for RGS14 activation of αMβ2. This result is consistent with previous studies which showed that the R333L point mutant did not interact with either Rap1, Rap2 (in yeast-two hybrid studies) [23] or H-Ras (in immunoprecipitation studies) [19]. As talin is considered to be the “final common step in integrin activation” [24], and since Rap1 acts upstream of talin, we wanted to confirm that RGS14 – as a Rap1 effector – acts upstream of αMβ2 in a talin-dependent manner. A plasmid expressing β2 defective in talin head association and activation (F754A), which is known to be the final step in integrin activation [4], was co-transfected with RGS14. RGS14 was unable to activate αMβ2(F754A) as measured by association with C3bi-RBC (Figure 1, inset; αMβ2(F754A) only, set at 100 c.f. αMβ2(F754A) + wild-type RGS14 = 95.1±0.2). This result was similar to talin1 co-transfected with αMβ2(F754A) in COS-7 cells, demonstrated here and shown previously [4,5] (Figure 1, inset; αMβ2(F754A) only, set at 100 c.f. αMβ2(F754A) + talin1 = 65.0±2.4 and [4]). This clearly suggests that RGS14 acts on αMβ2 in a talin1- and RBD1-dependent manner.

**Figure 2:**
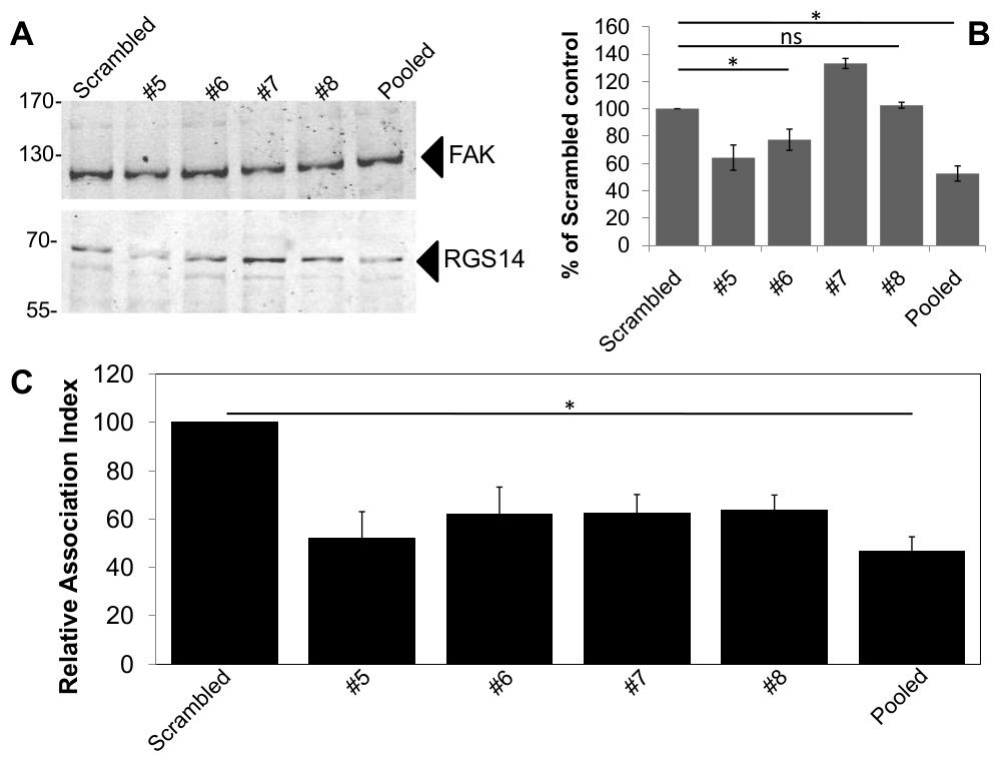
RGS14 is the Rap1 effector essential for RBC binding during αMβ2 activation. (**A**, **B**) J774.A1 macrophages were transfected with 60pmol of individual or pooled RGS14 or Scrambled siRNA as indicated and 5 days later, cells were analysed for RGS14 expression (**A**,**B**) and binding of C3bi-RBC (**C**). (**A**) Lysates of siRNA transfected J774.A1 cells were analysed by western blotting for the presence of RGS14 and focal adhesion kinase (FAK) as indicated. (**B**) Relative band intensities were determined as described in the Methods section, with the ratio of Scrambled siRNA and FAK intensities set to 100% for the negative control. (**C**) siRNA transfected J774.A1 cells were challenged with C3bi-RBC, processed for immunofluorescence and scored for RBC association, as described in the Methods section. Relative association indices were related to the values obtained from the negative (scrambled) controls and they are the mean ± S.D. of at least three independent experiments. Not significant (ns), *p*>0.05; *, *p*<0.05.

To confirm this result, varying doses (20–400pmol) of short interfering RNAs (siRNA) against human RGS14 or a control scrambled sequence were transfected into differentiated THP-1 human monocytes/macrophages. Transfected cells were left for 48hr before either challenging these cells with C3bi-RBC or lysing the cells for western blot analysis to determine levels of gene knock-down, with tubulin used as a loading control. However, we were unable to efficiently knockdown RGS14 in THP-1 cells (data not shown) so we used J774.A1 mouse macrophages transfected with 60pmol of either an individual or a pool of 4 siRNAs against mouse RGS14 siRNA. Gene silencing of RGS14 using individual siRNAs vary between 0-25% knock-down (#5, #6 and #8, Figure 2B), as determined by western blot analysis (Figure 2A, B). Knockdown of RGS14 using pooled siRNA was not complete with ~55% decrease in expression of RGS14 – when compared to the corresponding scrambled siRNA controls and focal adhesion kinase (FAK) loading controls (Figure 2A,B). This is reflected in a physiological effect as demonstrated with a decrease in bound C3bi-RBC – set at 100 (60pmol scrambled siRNA) c.f. 46.5±6.1 (pooled RGS14 siRNA) (Figure 2C). While there is a possibility of off-target effects, these findings and previous results with the overexpressed RGS14 gene (Figure 1) suggest a role for RGS14 in activating αMβ2 prior to particle binding and uptake. The modest decrease in bound C3bi-SRBC could be due to the presence of talin, a well documented activator of integrins, including αMβ2, on its β2 cytoplasmic tail [4,24]; kindlin-3, another activator of β2 class of integrins and shown to be involved in αMβ2-mediated spreading of αMβ2-expressing K562 cells [25,26]; and/or Rap1-interacting adaptor molecule (RIAM) which was recently demonstrated to regulate complement dependent phagocytosis, an αMβ2-mediated event [27], though the role of RIAM is still controversial [5]. Nevertheless, this suggests that RGS14 acts on αMβ2, either directly or in conjunction with the other integrin regulators described above. Interestingly, depletion of RGS14 led to a decrease in nerve- and fibroblast-growth factor induced neurite outgrowth in the neuronal cell line, PC12 [22] – a process generally thought to be driven by α3β1 integrins, specifically the cytoplasmic tail of the α3 integrin subunit [28,29].

**Figure 3:**
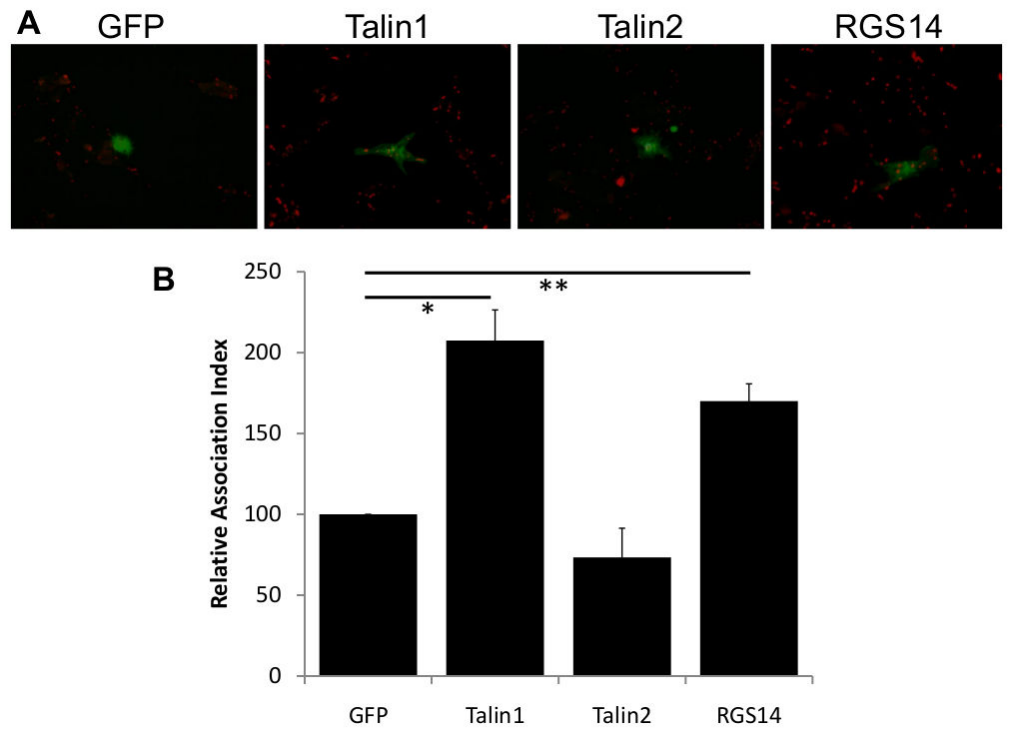
RGS14, but not talin2, is involved in the regulation of αMβ2 binding activity in macrophages. J774.A1 macrophages were transfected using magnetofection with either GFP-tagged talin1 or 2 or Flag-tagged RGS14 and 48 hr later, challenged with C3bi-RBC, processed for immunofluorescence using antibodies against Flag for cells expressing Flag-RGS14 plasmid (GFP-tagged proteins remained unstained) as well as antibodies to detect the C3bi-RBC (by anti-rabbit antibodies against the opsonising rabbit antibody) and analysed by epifluorescent microscopy (**A**) as described in the Methods section. (**A**) Representative examples; (**B**) cells were scored for RBC association, and indices are the mean ± S.D. of at least three independent experiments. *, *p*<0.001; **, *p*<0.05.

Finally, to confirm this RGS14/αMβ2 signalling mechanism, J774.A1 murine macrophages were transfected with either GFP as control, GFP-tagged talin1, talin2, or Flag-tagged RGS14. Although the transfection efficiency was low using magnetofection (<1%), cells remained viable and there were sufficient transfected cells (minimum of 25 cells) to allow analysis of phagocytosis (Figure 3). Consistent with previous data (Figure 1), J774.A1 cells transfected with RGS14 showed an increase in C3bi-RBC binding (170.0±10.7) compared to GFP control (set at 100, *p*<0.05), though not the same levels of binding as seen in the talin1-expressing control cells (207.5±18.9, *p*<0.001). Expression of talin2 did not increase binding of C3bi-RBC (73.3±18.1), further confirming that talin2 was not involved in activation of αMβ2. Studies showed that, unlike talin1, talin2 did not affect embryonic development, focal adhesion assembly or cell spreading [30,31]. However, talin2 is able to compensate the loss of talin1 in fibroblasts derived from talin1 knockout mice [32] and is possibly involved in the formation of actin-integrin links in mature muscle cells [33]. Interestingly, mouse testis and kidney express a talin head domain (N-terminal)-deficient isoform of talin2 [17] and it would be interesting to see if such a form of talin2 exists in macrophages. While we recognise the controversy surrounding RGS14 being Rap1 effector and interacting partner [22] and the lack of clarity regarding the function of talin2 in cellular models, we believe this study is a first step in delineating the pathway that governs αMβ2 activity. Interestingly, it has been shown recently that the Ras-binding domain (RBD) of RGS14 regulates the activity of RGS domains by interacting with, and enhancing their GTPase (GAP) activity [34]. This suggests the possibility of RGS14 acting in conjunction with Rap1. It also has to be noted that R-Ras, a Rap1 homologous small GTPase, like Rap1, also shares RGS14 as an effector [22]. R-Ras was previously shown to be required for αMβ2 activity during binding and phagocytosis of C3bi opsonised SRBC [3,35]. Therefore there is still a need to distinguish the role of Rap1 on RGS14 from R-Ras and how that regulates αMβ2 function, which would hopefully open up the field of integrin- and macrophage-biology.
